# Production of combustible fuels and carbon nanotubes from plastic wastes using an in-situ catalytic microwave pyrolysis process

**DOI:** 10.1038/s41598-023-36254-6

**Published:** 2023-06-03

**Authors:** Muhammad Irfan, Rishmail Saleem, Bilal Shoukat, Hammad Hussain, Shazia Shukrullah, Muhammad Yasin Naz, Saifur Rahman, Abdulnour Ali Jazem Ghanim, Grzegorz Nawalany, Tomasz Jakubowski

**Affiliations:** 1 Electrical Engineering Department, College of Engineering, Najran University Saudi Arabia, Najran, 61441 Saudi Arabia; 2 Department of Physics, University of Agriculture Faisalabad, Faisalabad, 38040 Pakistan; 3 Department of Agricultural Engineering, Faculty of Agricultural Engineering & Technology, University of Agriculture Faisalabad, Faisalabad, 38040 Pakistan; 4 Civil Engineering Department, College of Engineering, Najran University, Najran, 61441 Saudi Arabia; 5 Department of Rural Building, Faculty of Environmental Engineering and Land Surveying, University of Agriculture in Krakow, Al. Mickiewicza 24/28, 30-059 Krakow, Poland; 6 Department of Machine Operation, Ergonomics and Production Processes, Faculty of Production and Power Engineering, University of Agriculture in Krakow, 30-059 Krakow, Poland

**Keywords:** Environmental sciences, Energy science and technology, Engineering

## Abstract

This study performed in-situ microwave pyrolysis of plastic waste into hydrogen, liquid fuel and carbon nanotubes in the presence of Zeolite Socony Mobil ZSM-5 catalyst. In the presented microwave pyrolysis of plastics, activated carbon was used as a heat susceptor. The microwave power of 1 kW was employed to decompose high-density polyethylene (HDPE) and polypropylene (PP) wastes at moderate temperatures of 400–450 °C. The effect of plastic composition, catalyst loading and plastic type on liquid, gas and solid carbon products was quantified. This in-situ CMP reaction resulted in heavy hydrocarbons, hydrogen gas and carbon nanotubes as a solid residue. A relatively better hydrogen yield of 129.6 mmol/g as a green fuel was possible in this process. FTIR and gas chromatography analysis revealed that liquid product consisted of C_13+_ fraction hydrocarbons, such as alkanes, alkanes, and aromatics. TEM micrographs showed tubular-like structural morphology of the solid residue, which was identified as carbon nanotubes (CNTs) during X-ray diffraction analysis. The outer diameter of CNTs ranged from 30 to 93 nm from HDPE, 25–93 nm from PP and 30–54 nm for HDPE-PP mixure. The presented CMP process took just 2–4 min to completely pyrolyze the plastic feedstock into valuable products, leaving no polymeric residue.

## Introduction

Plastic products are ubiquitous in our daily lives. Because of their low cost, corrosion resistance, flexibility, durability, and lightweight, they are used in a variety of economic sectors, including residential, agricultural, automotive, commercial, medicine, packing materials, toys, demolition, and electrical equipment. High-density polyethylene (HDPE), polyethylene terephthalate (PET), low-density polyethylene (LDPE), polyvinyl chloride (PVC) and polypropylene (PP) were among the synthetic plastic polymers with the highest production rates over the past few years^1–3^. The applications of plastics are increasing with the increase in world population. Large-scale plastic production is raising several global concerns, including unsustainable production, environmental pollution and poor recycling processes or mechanisms^2^. Plastic waste management is essential for controlling environmental pollution at an acceptable level. Plastic polymers take decades to decompose and thus have an adverse impact on the environment. According to reports, waste plastic is the global 3^rd^ largest producer of landfills. Due to a significant increase in plastic wrapping industries, the production of plastic has expanded from 1.5 million metric tons in 1950, to 359 million metric tons in 2018 and around 367 million metric tons in 2020. Approximately 250 million metric tons of plastic waste are dumped in landfills and discharged into the atmosphere directly each year. Approximately 10 million tons are openly released into the oceans and a predicted 9–13% annual waste plastic growth might be created by 2050^3^. Waste plastics can discharge carcinogenic elements and other harmful compounds into landfills, contaminating groundwater. These toxic substances also reduce soil fertility. The marine ecology is also at risk from floating plastic debris in the ocean. Burning waste plastics produces dangerous emissions that are highly damaging to the environment when utilized as a direct energy source^4,5^.

Recycling plastic is challenging as removing many constraints of water pollution and other factors would be very expensive. Although recycling plastic would be able to minimize the amount of plastic waste, more consistent and maintainable methods are needed to convert the plastic waste to liquid oil, hydrogen gas fuel and CNTs^5^. The treatment of waste plastic has become a major problem, and pyrolysis is a tertiary chemical process that quickly transforms plastic waste into carbon and hydrogen fuel by thermally breaking down long-chain polymer molecules into smaller ones in an oxygen-free environment. The factors of pyrolysis products, such as temperature, catalyst type, residence time, pressure, reactor type, particle size, and fluidizing gas, all affect the quantity and quality of the product. It is possible to obtain desired valuable items by adjusting a number of parameters. For instance, the maximum liquid was produced during the pyrolysis of LDPE at 550 °C and PET at 520 °C. In order to generate desired products, the catalyst-plastic mixture, heat conversion, and reaction efficiency must all be carefully considered in the reactor design. The biomass and plastic waste are broken down using batch, continuous, or semi-batch reactors, conical spouted beds, fluidizing beds, and other similar geometries^6^.

Pyrolysis via microwave heating is a novel and promising technique for recycling waste plastic and biomass into fuel. Microwave-initiated pyrolysis process requires a dielectric substance to absorb microwaves. In the pyrolysis of plastics, caron back or activated carbon was used as a microwave susceptor, transforming microwave energy into thermal energy by changing the dipole alignment of polar molecules. The long-chain hydrocarbons of raw materials are broken into short-chain molecules through rapid microwave heating with high product selectivity^7^. A temperature in the range of 350–650 °C is required to disintegrate the polymer/biomass long-chain molecules into low molecular weight compounds in an oxygen-free environment because combustion of the product does not happen in an oxygen-free environment. Thermal decomposition of biomass produces combustible gases and charcoal. Most of the gases may be condensed into a combustible liquid known as pyrolysis oil, while some permanent gases like H_2_, CO, CO_2_ and light hydrocarbons are also observed in the process^8^. Microwave heating has many advantages as compared to conventional heating. This method ensures specific heating and higher efficiency of energy conversion. So, this technique is most appropriate for degrading materials containing biomass, brown coal, wood, and plastic waste^9^. Catalytic microwave pyrolysis also improves the polymer conversion rate, product distribution and yielding by reducing the process time and operating temperature. Microwave radiations target the material by heating the core of the material. Because of their acidic nature and porous structure, zeolites and metal oxide catalysts have been shown to be the most energetic catalysts. By modifying the characteristics of zeolites, suitable liquid and gaseous products can be obtained^10^.

A catalyst is required for catalytic pyrolysis to accelerate the reaction and convert plastic wastes into liquid oil at relatively low temperatures. Various types of catalysts are used, such as Fluid Catalytic Cracking (FCC), Zeolite Socony Mobil-5 (HZSM-5, ZSM-5), Natural Zeolite, Cu-Al_2_O_3,_ and Red Mud. Chen et al.^11^ produced a magnetic catalyst from diaper waste to catalyze the reaction to produce biodiesel. They reported that this catalyst is highly efficient, environmentally friendly and low-cost compared to other catalysts. Wang et al.^12^ derived porous carbon from potassium citrate for CO_2_ capture and dye adsorption applications. In pyrolysis, the catalyst drives the rate of cracking, which results in a rapid increase in gas production with decreased liquid oil. In this study, the reactivity of zeolite catalysts on feedstock in MAP was studied. A catalytic fixed bed reactor was utilized in the ex-situ procedure to increase the activity of the catalyst. The microwave-assisted pyrolysis of Douglas fir in the presence of ZSM-5 has produced aromatic and phenol- range hydrocarbons^13^. Microwave pyrolysis of HDPE using ZSM-5 catalyst has produced 47.4% yield of liquid and 24.5% of waxes at 560 °C^14,15^. In response to these findings, in-situ microwave pyrolysis of polyethylene and polypropylene into hydrogen, liquid fuel, and carbon nanotubes was carried out in the presence of a Zeolite Socony Mobil ZSM-5 catalyst.

## Materials and methods

**Figure 1:**
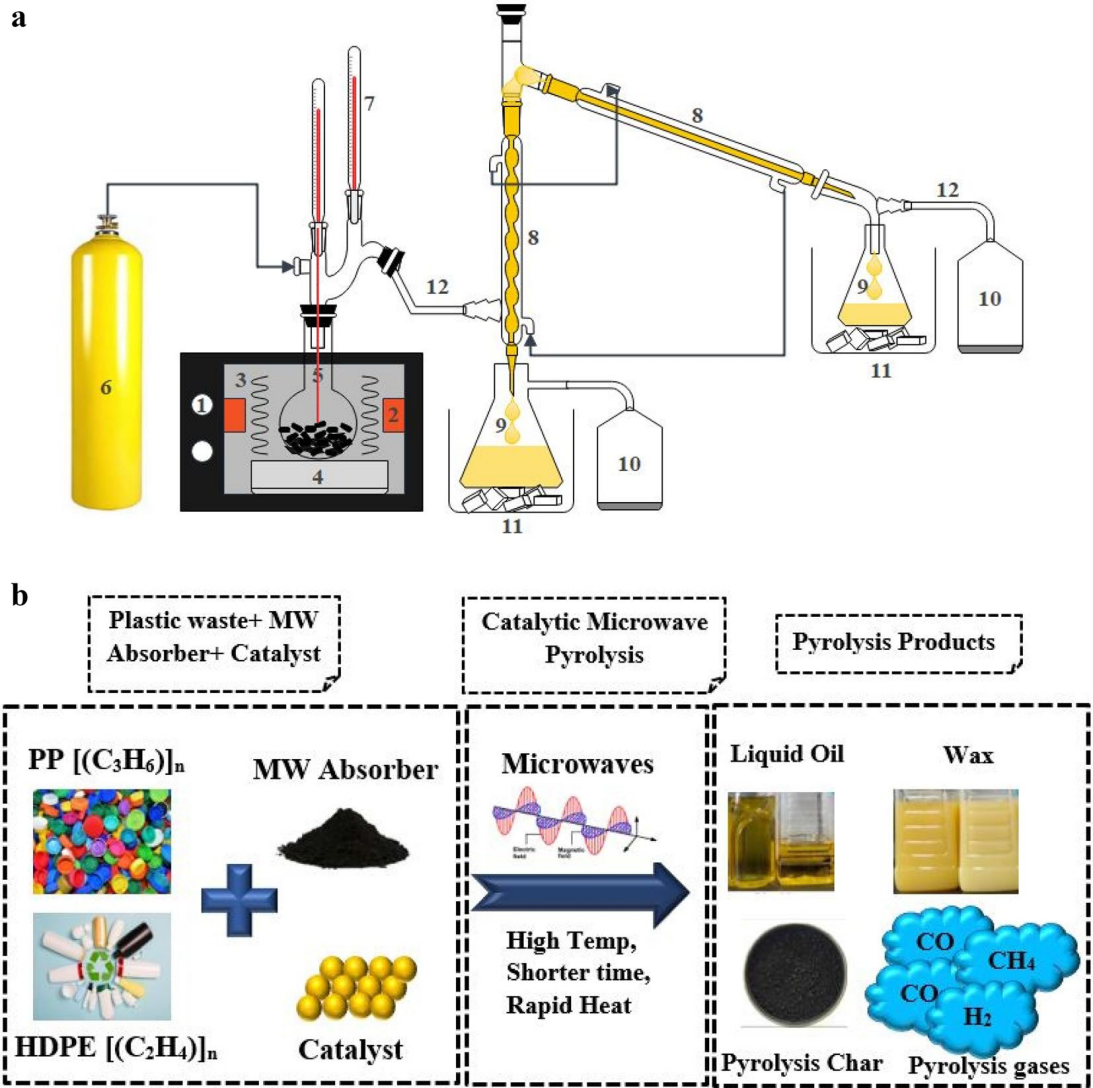
(**a**) Schematic of pyrolysis setup: (1) microwave reactor; (2) waveguide; (3) microwaves; (4) ceramic fibred block; (5) pyrolysis reactor; (6) nitrogen gas inlet; (7) thermometer; (8) Liebig condensers; (9) oil collecting flasks; (10) gas sampling; (11) cold traps; (12) connecting tubes. (**b**) Mechanism involved in destruction of plastic into liquid fuel and gaseous products through catalytic microwave pyrolysis.

The pure and mixed forms of HDPE and PP wastes were used in the microwave pyrolysis experiments. The plastic feedstock was recovered from household plastic bottles, shampoo bottles and food containers. The main characteristics and composition of plastics and their mixture are given in Table 1. The plastics were washed, dried and chopped into 5 mm pieces and fed into the pyrolytic reactor, as shown schematically in Fig. 1a. ZSM-5 catalyst of analytical grade was purchased from a chemical store and used to convert plastic waste hydrocarbons into diesel and gasoline range hydrocarbons and syngas. ZSM-5 is a microporous, 3D synthetic zeolite that contains alumina (Al) and silica (Si), with silica content notably higher than alumina. The chemical composition of the catalyst is: Silicon Oxide-33%, Aluminum Oxide-28%, Sodium Oxide-17% and water-22%. With Si/Al ratio of 40, the catalyst had a pore diameter of 5 Å, surface area of 397 m^2^/g and pore volume of 0.178 cm^3^/g. The catalyst was activated by calcining it at 500 $^\circ{\rm C}$ for 2 h. The activated carbon worked as a heat susceptor to initiate the pyrolysis process by absorbing microwave energy in the presence of ZSM-5 catalyst. The heat susceptor converted microwave radiations into thermal waves, decomposing the plastic waste into high-value hydrocarbons, as explained in Fig. 1b.

**Table 1:** Elemental and proximate analyses of HDPE and PP feedstocks.

Plastic type	Molecular weight (Kg/mol)	HHV(MJ/kg)	Proximate analysis (wt.%)				Elemental analysis (wt.%)		
			Moisture	Volatile Matter	Fixed Carbon	Ash	C	H	N
HDPE	130–300	47.22	0.0	99.1	0.1	0.8	85.5	14.4	0.0
PP	75–200	46.68	0.1	99.3	0.5	0.1	85.3	14.3	0.0

The catalytic microwave pyrolysis setup was built up by modifying the household microwave oven into a microwave pyrolysis reactor working at 1 kW power, with temperature ranges from 400 to 450 °C , provided microwaves of frequency 2450 MHz from the waveguide shown in Fig. 1a. There were two thermocouples: one for monitoring the vapor temperature and 2nd for monitoring the pyrolysis process temperature. The later thermocouple was in contact with the feedstock. A microwave source, consisting of magnetons, was operated at 100 °C to produce microwaves. Magneton functioning at 100 °C refers to the magnetron temperature when it generates microwaves to heat the feedstock in the temperature range of 400–450 °C. The apparatus temperature is 100 °C, while the microwave process temperature is 400–450 °C. The focused microwaves pyrolyze the waste plastic by changing the dipole alignment of polar molecules. The nitrogen was added to the reactor cavity at a flowrate of 1.5 L/min to create the working environment for the pyrolysis process. The ceramic fibred block was placed below the pyrolytic reactor to minimize the heating loss. Condensers, cold traps, oil collecting flasks and gas analyzer were used to condense and maintain the pyrolytic vapors flow and separate them into liquid and gas fuels. The liquid oil was collected into flasks and gas was analyzed using a gas analyzer.

In this catalytic microwave pyrolysis process, 50 g of HDPE and PP were chopped into 5 mm pieces. The plastic feedstock, ZSM-5 catalyst and activated carbon with a ratio of 10:1 was directly faded into the pyrolytic reactor. The mixture of plastic wastes (HDPE and PP), ZSM-5 catalyst and activated carbon reacted and produced the pyrolytic vapors, which passed through the condensation apparatus, and separated into valuable products. A cold trap consisted of two condensers and a water-cooling pump to cool and control the flow of liquid oil. The solid residue obtained at the end of the process was collected from the reactor for further analysis. The solid residue at the end of the pyrolysis process was identified as multi-walled CNTs. The yield of liquid oil products was calculated by weighing the collecting flasks after the pyrolysis process. The residue yield was estimated by comparing the weights of the pyrolysis pot at the start and end of the pyrolysis process.

## Results and discussion

### Microwave pyrolysis mechanism

**Figure 2:**
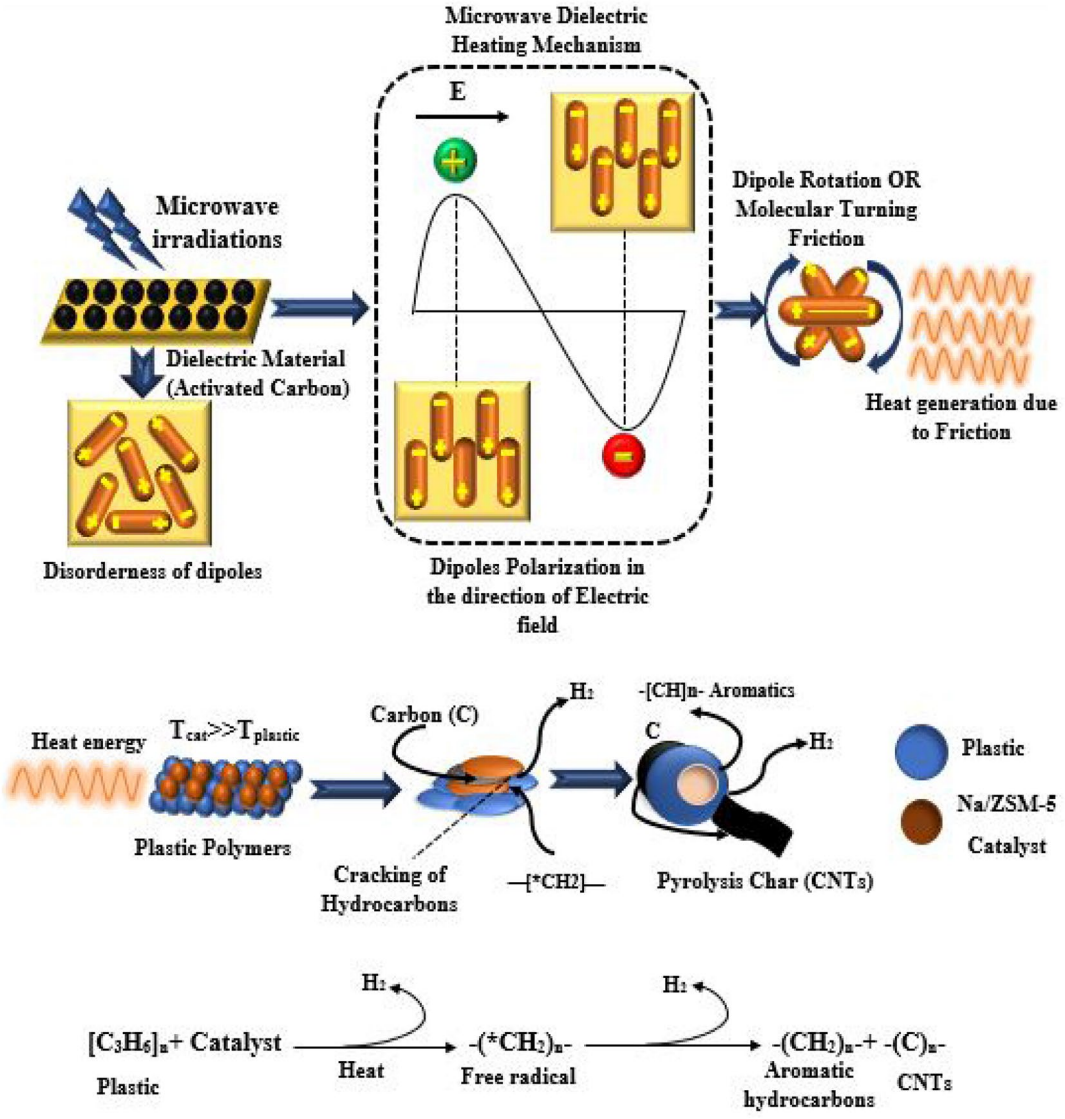
Mechanism of interaction of microwave radiations with dielectric for pyrolysis of waste plastic in the presence of Na/ZSM-5 catalyst.

The interaction of microwaves with material and conversion of microwaves into heat energy for decomposition of plastic waste into useful products is illustrated schematically in Fig. 2. In the case of microwave pyrolysis, a dielectric material is used as a microwave absorber or susceptor. We used activated carbon as a dielectric substance that absorbed the microwaves and converted them into heat energy. In an interaction between microwave radiations and dielectric material, the electric field directly interacts with the charged particles (e^-^) of the materials to generate heat, which causes the dislocation of charged particles from their equilibrium positions^4^. It involves two mechanisms: dipole polarization and dipole rotation. In dipole polarization, the permanent or induced dipoles of the molecules tend to align in the direction of the oscillating electric field.

In dipole rotation, the polar molecules rotate back and forth continuously to align the dipole in a fluctuating electric field. The reorientation between rotating molecules in both mechanisms results in friction, which causes heat generation^5^. The generated heat is highly selective and rapidly interacts with plastic waste when sodium zeolite is used as a catalyst. The long-chain hydrocarbons of plastic were cracked and converted into shorter-chain hydrocarbons of aromatics or alkenes (wax) with the evolution of hydrogen gas as a fuel and solid carbon residue.

### Effect of plastic type on products

**Figure 3:**
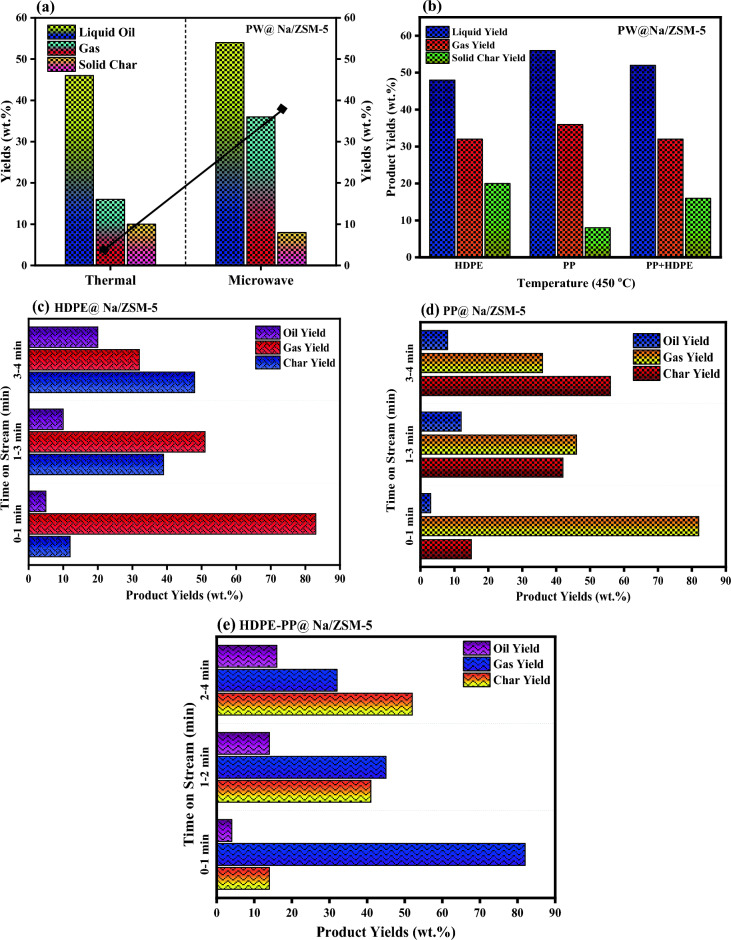
(**a**) A comparison on product yield obtained via thermal and microwave pyrolysis methods, (**b**) effect of plastic type on product yield in the presence of Na/ZSM-5 catalyst at 450 $^\circ{\rm C}$, (**c**) time-on-stream evaluation of product yield from HDPE, (**d**) PP, and (**e**) HDPE-PP.

Figure 3a provides a comparison of product yield obtained via thermal and microwave deconstruction of plastic waste. The microwave pyrolysis was performed in an oxygen-free environment in the temperature range of 4000–450 °C. On the other hand, thermal pyrolysis process takes several hours to complete even at higher temperatures. In the microwave pyrolysis process, high liquid and gas yields were obtained within 24 min at relatively lower temperatures (400–450 °C) in the absence of oxygen^16–19^. Compared with the thermal decomposition of plastic waste, the yield of solid char also decreased in the microwave pyrolysis process. About 85–95% of feedstock was successfully converted into valuable products. In the microwave pyrolysis process. The desirable products can be obtained by manipulating the pyrolysis parameters i.e. retention time, catalyst type, temperature, and feedstock-to-catalyst ratio^18,19^. Figure 3b demonstrated the effect of plastic-type on the product yield at 450 °C temperature in the presence of Na/ZSM-5 catalyst. The graph illustrated that the yield of liquid oil, obtained from HDPE, PP and mixture (HDPE-PP), was about 56%, 48% and 42%, respectively. Similarly, the gas that evolved from these plastic wastes was about 24%, 40% and 42%, respectively. The char residue obtained from HDPE and mixture was maximum as compared to PP^20^. The gas contained a high concentration of hydrogen and some concentration of other gases like methane, carbon monoxide and carbon dioxide. Various types of catalysts are used to improve the composition and distribution of pyrolysis products, such as gas fuel and liquid oil. The presence of catalysts affects the pyrolytic temperature as well. The catalyst accelerates the reaction, transforming waste plastic into valuable products^21^.

### Catalysis effect

Figure 3c–e shows the product yield distribution and composition obtained from HDPE, PP and mixture (HDPE-PP) over time. To study the effect of catalyst on product distribution, feedstock in 10:1 ratio with catalyst was pyrolyzed in a microwave reactor at 450 °C temperature. The time-on-stream evolution of product distribution in 4 min was divided into 3 steps during the process. In 1st step, the yield of gas (vapors) produced from the process increased from 80 to 88%, solid residue from 10 to 15%, while a negligible amount of liquid oil was observed. In the 2nd step, the gas yield decreased from 88 to 50%, char residue (waxes) increased from 30 to 40%, and liquid oil slightly increased. In 3rd step, the liquid oil, and gas yields approached to 48% and 24%, respectively and solid char reduced to 15% in contrast to other stages of time-on-stream evaluation on product yield^14,22^.

Table 2 shows the influence of different pyrolytic parameters on the yield of liquid oil, gas and solid residue. HDPE undergoes 80% conversion into valuable products, while the conversion of PP and HDPE-PP mixture was 88% and 84%, respectively. The conversion of feedstock into useful products was estimated using Eq. (1). Similarly, liquid, gas, char yield, and energy reconvey in the form of liquid oil were calculated using Eqs. (2–7)^23–25^.

**Table 2:** Effect of different pyrolysis parameters on product yield.

Parameter	HDPE	PP	(PP + HDPE)
Catalyst	Na/ZSM-5	Na/ZSM-5	Na/ZSM-5
MW susceptor	Activated carbon	Activated carbon	Activated carbon
Plastic: catalyst	10:1	10:1	10:1
MW power	1KW	1KW	1KW
Temperature	450 °C	450 °C	450 °C
Mass of feedstock (g)	50 g	50 g	50 g
% Conversion	80%	88%	84%
Liquid oil yield (wt.%)	56%	48%	52%
Gas yield (wt.%)	24%	40%	32%
Solid char (wt.%)	20%	12%	16%
% Energy recovery in Oil	51.28	44.35	47.84
Density of liquid Oil (g/cm^3^)	0.822	0.816	0.825
HHV of liquid Oil (MJ/Kg)	42.332	43.138	42.735
HHV of plastics (MJ/Kg)	46.22	46.68	46.45

1 $$\% Conversion= \frac{M-M_2}{M}\times 100$$

2 $$Liquid yield \left(Lwt.\%\right)=\frac{{M}_{1}}{M}\times 100$$

3 $$Char yield \left(Cwt.\%\right)=\frac{{M}_{2}}{M}\times 100$$

4 $$Gas yield \left(Gwt.\%\right)=100-\left(Lwt.\%+Cwt.\%\right)$$

5 $$\rho \left(liquid oil\right)=\frac{{M}_{1}}{{V}_{1}}$$

In this equation, *M* is mass of plastic (g), *M*_*1*_ is liquid mass (g), *M*_*2*_ is solid residue (g), *V*_*1*_ is the volume of liquid oil in ml, and $\rho$ is the density of liquid oil (g/cm^3^).

6 $$\% Energy \,recovery\, in \,liquid \,oil=Lwt.\% \times \frac{{HHV}_{oil}}{{HHV}_{plastic}}$$

The HHV of a fuel is the amount of energy released by a specific quantity of feedstock at the initial temperature once it is decomposed. The HHV of oil from the pyrolysis of plastic waste was calculated depending on elemental analysis by using the following equation^14^:

7 $$HHV\left( {{\raise0.5ex\hbox{$\scriptstyle {MJ}$} \kern-0.1em/\kern-0.15em \lower0.25ex\hbox{$\scriptstyle {kg}$}}} \right)~ = ~\frac{{\left( {34C + 124.3H + 6.3N + 19.3S - 9.8O} \right)}}{{100}}$$

In this equation, O = Oxygen, H = Hydrogen, C = Carbon, S = Sulphur and N = Nitrogen.

### Gaseous product yield

**Figure 4:**
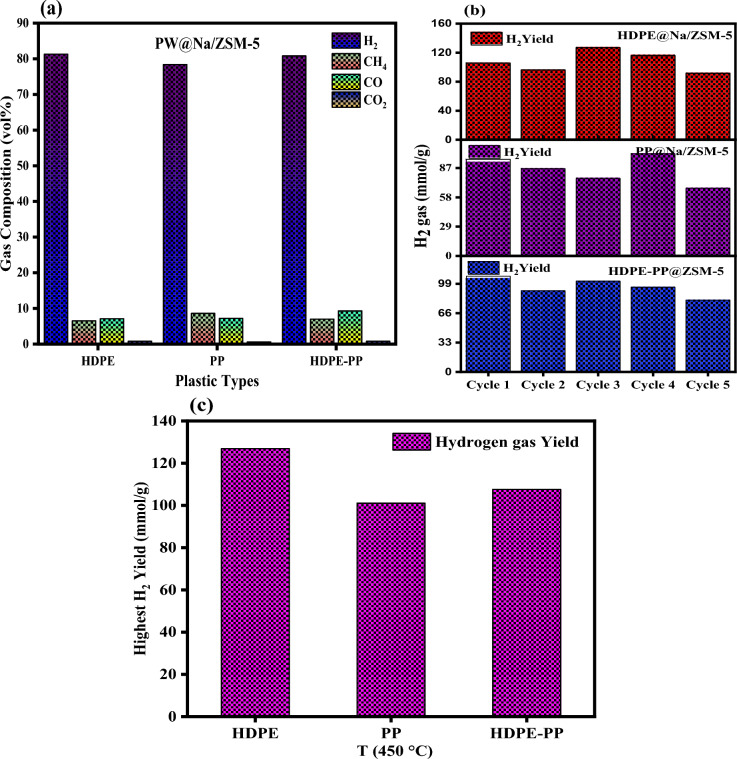
(**a**) Different gas compositions of H_2_, CH_3_, CO, and CO_2_ over plastic type, (**b**) hydrogen gas efficiency (mmol/g) for each cycle of experiment for different plastic wastes, and (**c**) highest gas yield efficiency in different plastic wastes at 450 °C.

Figure 4a reports volume percentage of H_2_, CH_3_, CO, and CO_2_ evolved from catalytic microwave pyrolysis of HDPE, PP, and their mixture (HDPE-PP). The H_2_, CH_3_, CO composition of HDPE, PP, and HDPE-PP was measured about 78–81%, 6–8%, 7–8%, respectively. Figure 4b demonstrates the hydrogen gas yield (mmol/g) distribution evolved from HDPE, PP, and its mixture over several successive cycles in each experiment. In HDPE decomposition, the highest hydrogen gas evolved in 3rd cycle in contrast to other cycles, typically between 91.7 and 126.9 mmol/g. In PP pyrolysis, the maximum amount of hydrogen gas was observed in 4th cycle in the range of 66.9–101.1 mmol/g. In microwave pyrolysis of HDPE-PP, the maximum hydrogen gas composition was detected in 3rd cycle in the range of 88.8–107.6 mmol/g. The highest hydrogen gas efficiency was measured during microwave pyrolysis of HDPE, PP, and HDPE-PP at 450 °C (Fig. 4c). The maximum hydrogen gas that evolved from HDPE during the pyrolysis process was 126.9 mmol/g, while in PP, and HDPE-PP pyrolysis, the evolved gas was 101.1 mmol/g and 107.6 mmol/g, respectively.

The probe of a gas analyzer was injected into the microwave reactor to check the gas efficiency after every 30 s of the pyrolysis process^26^. The gas analyzer was used to calculate the volume and composition of gases, from which the masses of each gas can be calculated. The following Eqs. (8–10) calculate the yield of obtained gas. The H_2_ yield is calculated by dividing the number of moles of H_2_ by the total mass of plastic utilized. The efficiency of H_2_ was determined by dividing the mass of H_2_ present in all gases by the theoretical amount of H_2_ contained in plastic^27^.

8 $$Gas\, Yield\, (wt.\%)= \frac{{m}_{g}}{{m}_{p}}\times 100\%$$

9 $$Hydrogen\, Yield\, (mmol/{g}_{P})=\frac{{m}_{{H}_{2}}}{{m}_{P}}$$

10 $$Hydrogen\, Efficeincy(\%)=\frac{{m}_{{g}_{H}}}{{m}_{Th}}$$

where *m*_*p*_ is plastic waste mass, m_g_ is gas amount, mH_2_ is the moles of hydrogen, *m*_*gH*_ is the total hydrogen mass present in the gaseous product and *m*_*Th*_ is the theoretical hydrogen mass present in plastic. Table 3 demonstrates the gas composition (vol%) evolved from microwave pyrolysis of HDPE, PP, and mixture (HDPE-PP) with feedstock to catalyst ratio of 10:1 at 450 °C. The gas evolved from this process mainly contained a high value of H_2_, and some amount of CH_3_, CO, and CO_2_ and other impurities.

**Table 3:** Experimental results of gas composition (vol%) evolved from microwave pyrolysis of waste plastics at 450 °C.

Gas	HDPE	PP	HDPE-PP
*Gas composition (Vol%)*			
H_2_	81.3	78.4	80.8
CH_4_	6.52	8.6	7.0
CO	7.1	7.2	9.3
CO_2_	0.8	0.8	0.8
H_2_ gas (mmol/g)	126.9 mmol/g	101.1 mmol/g	107.6 mmol/g

### Characterization of liquid and solid products

**Figure 5:**
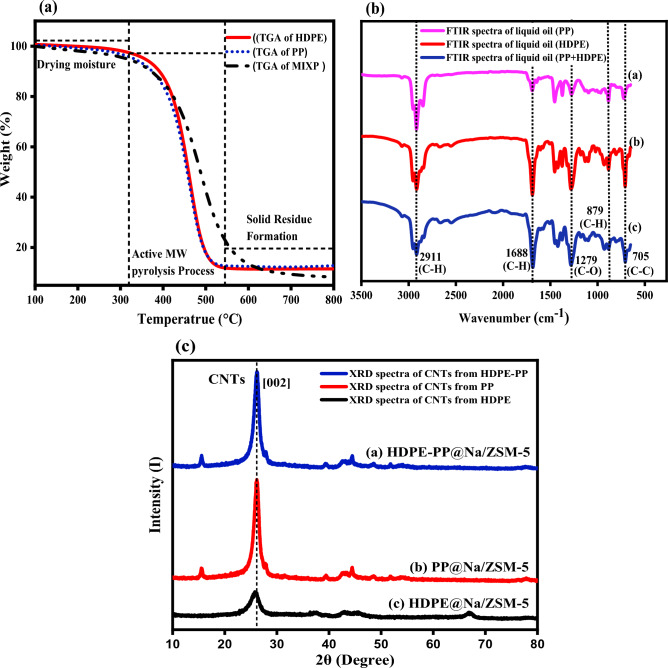
(**a**) Thermogravimetric curves of different waste plastics, (**b**) FTIR of oil obtained from microwave pyrolysis of different plastic wastes, and (**c**) X-ray diffraction spectra of solid residue in the form of CNTs.

TGA is an analytical technique used to determine the thermal stability of a material as well as the amount of volatile chemicals present in the sample under investigation. Figure 5a shows TGA curves of HDPE, PP, and HDPE-PP feedstocks in the range of 100–800 °C. Because the plastic material structure lacks inherent water, thermal degradation occurs at higher temperatures and took less time^28^. As the temperature of the plastic mixture rises from 100 to 300 °C, the material first melts and moisture present in the plastic is dried, but a further increase in temperature from 320 to 540 °C causes the chemical bonds to break. Due to the similarity of the chemical linkages in the molecular structures of HPDE and PP, the degradation of HDPE-PP revealed only one peak in the temperature range of 300–450 °C.

Instead of burning, the active microwave pyrolysis process occurs when the sample is heated under a nitrogen environment in the absence of air. As a result, TGA testing environment would be exactly similar to that of a pyrolysis reactor. Thermal degradation begins with an increasing slope for all samples. The slope of the graph diminishes after passing through an extreme and ultimately becomes horizontal, where char production occurs^29^. The TGA profiles were produced in the temperature range of 100–800 °C. It has been discovered that mixed waste plastic degrades faster than separate plastics, resulting in a synergistic effect. The plastic degradation started after 300 °C and no degradation or weight loss was observed after 600 °C. Any changes in weight at temperatures below 300 °C, also called initial temperature, were due to removal of moisture content or drying of the sample. Volatile material was found in the range of 300 to 600 °C. The residue remains unchanged after 600 °C.

The CNTs from PP waste were made up of multiple components with degradation temperatures range of 400–550 °C. Each polymer showed a single phase breakdown into CNTs as represented by a single peak. The TGA curve showed that HDPE started degrading at temperatures near 350 °C and finally degraded at a high temperature of 600 °C. HDPE has a highly linear structure. PP is made up of repeated units that have methyl groups on a core carbon chain as a side chain. As a result, the PP degradation temperature was lower than the HDPE degradation temperature^30^. The TGA curve for a mixture of HDPE and PP showed a high linear degradation curve. Table 4 gives the initial weight loss and oxidation temperature of different plastic wastes pyrolyzed via the microwave pyrolysis method.

**Table 4:** Initial weight loss and oxidation temperature obtained from TGA curves.

Plastic types	Initial weight loss T ($\boldsymbol{^\circ{\rm C} }$)	Oxidation temperature ($\boldsymbol{^\circ{\rm C} }$)
HDPE	338	553
PP	346	530
Mixture (HDPE + PP)	328	580

FTIR spectra and chemical compounds with functional groups of hydrocarbons in oil obtained from the catalytic pyrolysis of HDPE, PP and HDPE-PP are given in Fig. 5b. FTIR spectra showed typically similar results for oils obtained from different plastic wastes with slightly varying peak intensities. The classification of chemical compounds and functional groups of oil products are given in Table 5. The FTIR peaks, ranging from 3000 to 2840 cm^-1^, revealed high intensities corresponding to asymmetric C–H stretching bands. These bands revealed the presence of alkanes in the hydrocarbons of oils. The FTIR peaks in 1683–1286 cm^-1^ range represent alkenes or aromatic rings C–O and C–H stretching bending vibration in the sample. The peaks in the range of 970–897 cm^-1^ revealed C–H bending vibration and fingerprints of alkenes. The absorption peaks observed in the 800–690 cm^-1^ region showed single-ring aromatic compounds. These peaks confirmed C–H out-of-plane bending in the compounds of oil^31,32^. The obtained oils were rich in alkanes and alkenes like aliphatic hydrocarbons. They also contained a small amount of single-ring aromatic hydrocarbons, ketones, and aldehydes. FTIR spectra of liquid fuel were also compared with diesel and heavy gasoline range hydrocarbons. The high-intensity peaks of aliphatic compounds were similar to diesel fuel, which had the potential to convert plastic wastes through microwave pyrolysis into valuable heavy liquid fuel (waxes) that would be upgraded by hydrogenation method into light-weight diesel fuel^33,34^.

**Table 5:** Classification of chemical compounds and functional groups of oil products.

Wavenumber (cm^-1^)	Functional groups	Compound
3000–2840	Asymmetric C–H stretching bands	Alkanes
1683–1286	C–O and C–H stretching bending vibration	Alkenes
970–897	C–H bending vibration	Alkenes
800–690	C–H out of plane bending	Single-ring aromatics

XRD patterns of CNTs product of microwave pyrolysis of HDPE, PP and HDPE-PP feedstocks are reported in Fig. 5c. XRD peaks at 2$\theta$ of 26.1$^\circ$, 39.3$^\circ$, and 44.4$^\circ$ were assigned to (002), (100), and (101) diffraction planes, respectively. A sharp characteristic peak of (002) plane appeared in all samples, indicating the graphite structure of CNTs. The intensity and FWHM of this peak are interlinked with interplanar d-spacing and lattice parameters of the material^35,36^. The interplanar space (d_002_) increases with the process temperature. The large value of FWHM indicated that CNTs have more defects, resonance, and distortion. Relative intensities of diffraction peaks deduced the position of the atoms within a unit cell compared with the most intense peak (002) of XRD pattern^37,38^. The interplanar space (d_002_) of intense peak (002) indicated the maximum microwave catalytic conversation of plastic into CNTs compared with the other two peaks^39,40^. The relative intensities of planes of (120) and (202) were compared with sharp peak (002) intensity, which deduced the position of the atom in a unit cell of hexagonal crystal structure of CNTs. The low interplanar d-spacing (0.34) showed a high crystalline structure of CNTs. The crystalline size, relative intensity, and other parameters depending on XRD were calculated using Bragg’s law and the Scherrer equation. The findings of XRD analysis are reported in Table 6. The broadening of diffraction peaks in XRD patterns was analyzed to calculate the in-plane crystallite size of CNTs. The curved wall of CNTs affects their diffraction patterns and, consequently the crystallite size. The Scherrer equation for relating crystal edge length to peak breadth was used in these calculations:

11 $$L=\frac{K\lambda }{\Delta \left(2\theta \right)cos\theta }$$

where θ in this equation is a diffraction angle, λ wavelength of the diffraction, K is a Scherrer constant, and Δ(2θ) is line broadening due to crystallite size. Since scattering angles were sufficiently small, the approximation $cos\theta \approx 1$ was used in these calculations. The crystalline size was calculated using (002) and (110) peaks. The interplanar spacing (d_002_) was calculated using Bragg’s law:

12 $${\text{d}}\left( {\text{\AA}} \right) = \frac{\lambda }{2\sin \theta }$$

where $\lambda$ is the wavelength X-rays, d(Å) is interplanar spacing.

**Table 6:** XRD analysis of CNTs produced through pyrolysis of plastic waste.

Samples	Pos. (2 ${\varvec{\theta}}$)	d_002_ (nm)	Crystalline size (nm)
HDPE	25.8 $^\circ$	0.344	31.75 nm
PP	26.2 $^\circ$	0.339	58.1 nm
HDPE-PP	26.1 $^\circ$	0.342	59.3 nm

### Morphology of CNTs

**Figure 6:**
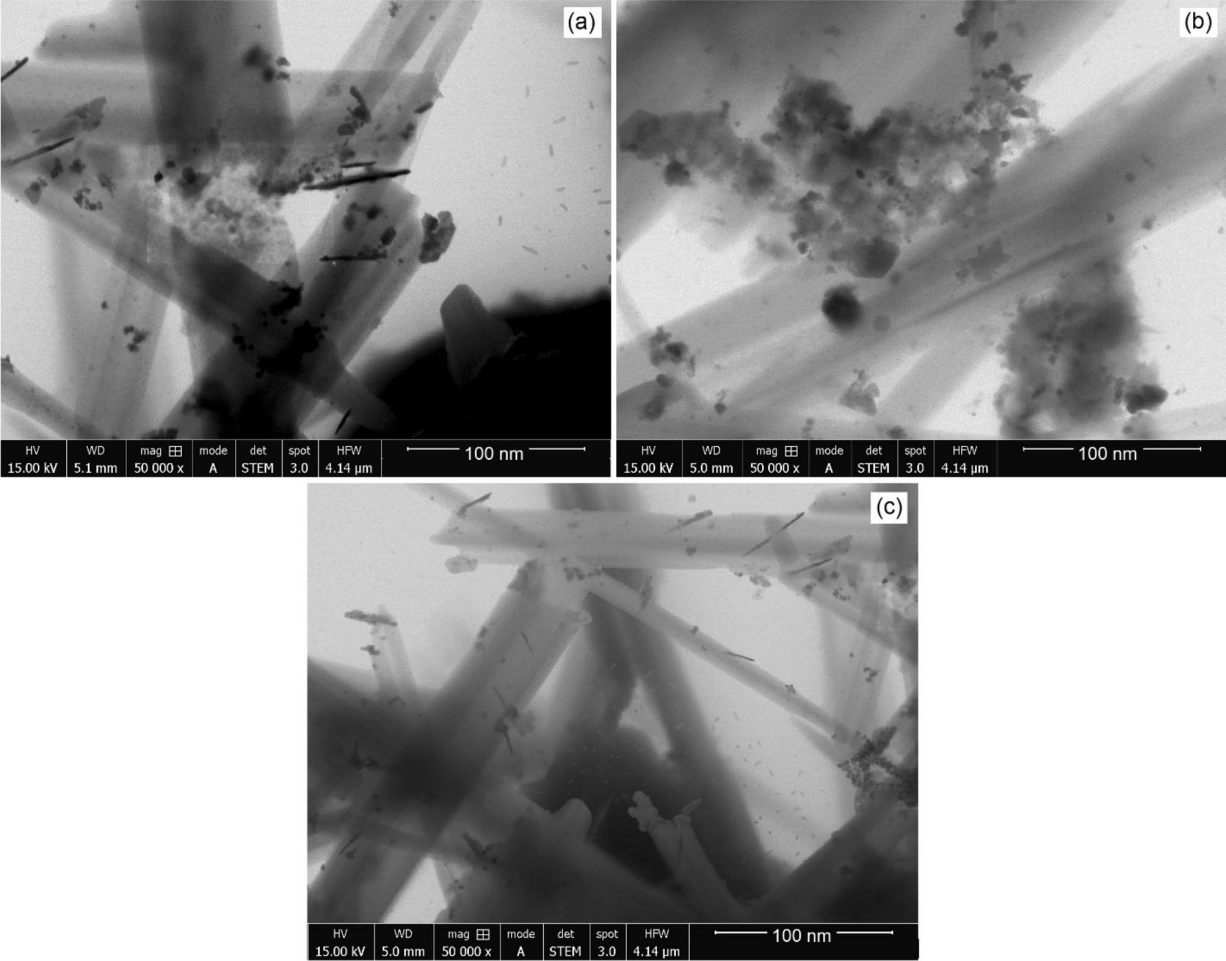
STEM images of CNTs produced during pyrolysis of HDPE (**a**), PP (**b**), and HDPE-PP (**c**).

STEM images of the solid product of catalytic pyrolysis of HDPE, PP and HDPE-PP are shown in Fig. 6. Since STEM images are not as clear as TEM images, these images provide a rough understanding of the internal structure of the nanotubes. The STEM images revealed multi-walled structures of CNT product. All CNTs samples contained some catalyst particles and residual impurities. Significant growth of CNTs during microwaves assisted catalytic cracking of pure and mixed plastic wastes over ZSM-5 catalyst. A typical hexagonal morphology with a clinoptilolite phase of ZSM-5 catalyst and needle-like morphology with a mineralogical phase of CNTs was observed. These CNTs correspond to (002) plane, showing better crystallinity of tube structure^41,42^. The outer diameter of CNTs ranged from 30 to 93 nm from HDPE, 25 nm to 93 nm from PP and 30 nm to 54 nm for HDPE-PP mixture.

The mixed waste produced the narrowest diameter of CNTs. The structures were also in more developed form with uniform morphology as compared to pure HDPE and PP. The catalyst particles are also smaller and lower in number in CNT structures from mixed plastic waste. Since large number of branched chains in PP facilitate fast decomposition of pure PP molecules, it is challenging to grow well-structured CNTs due to fast cracking of chains in PP. On the other hand, decomposition of pure HDPE molecules is assumed to be slower than PP. HDPE contains unbranched carbon chains, which make it difficult to decompose at a fast rate. The decomposition of pure HDPE also results in a more complex residue than pure PP. Therefore, HDPE-produced CNTs pose more defects and rugged surface morphology due to low degree of graphitization. Having a good mix of unbranched and branched carbon chains, HDPE-PP mixture undergoes decomposition at moderate rates allowing the formation of well-structured nanotubes. The CNT structures, produced with pure plastic wastes, contained a large number of agglomerated catalyst particles and amorphous carbon^43^. The entrapped impurities and catalyst particles can clearly be seen STEM images in Fig. 6. Wang et al.^44^ conducted catalytic pyrolysis of plastic waste for the production of valuable carbon in an effort to convert waste materials into and energy and valuable materials. They tested Ni/cordierite, Fe/cordierite and Ni-Mg/cordierite catalysts to pyrolyze polypropylene plastic. The Ni/cordierite catalyst results in the highest filamentous carbon yield of 93%. The strong metal-support interaction inside the Ni-Mg-based catalyst inhibited CNT growth, resulting in CNTs that were shorter in length and bigger in diameter (about 30–50 nm). Most of the tubes were found in irregular cylindrical shapes.

### Chromatography analysis of liquid product

**Figure 7:**
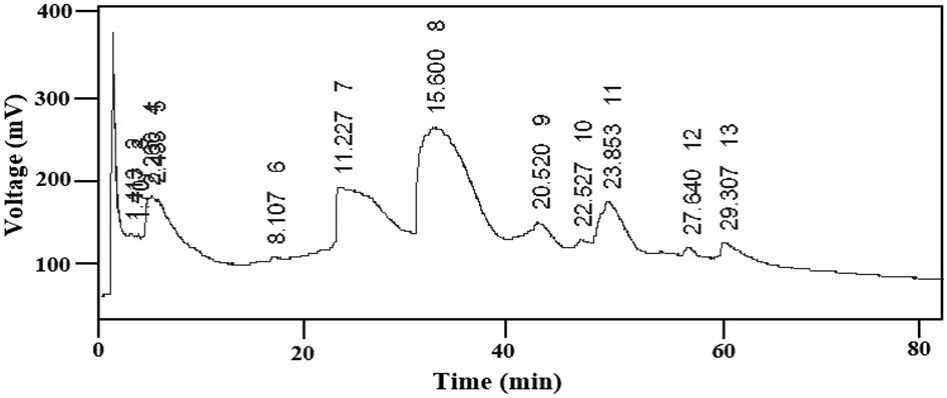
A typical GC–MS profile of liquid product obtained during microwave pyrolysis of plastic waste.

The chemical composition and main components of liquid products of microwave pyrolysis of HDPE, PP and HDPE-PP were identified using gas chromatography analysis. The liquid product was initially dissolved in methanol to determine its chemical composition. A typical GC–MS profile of the composition of the liquid product is given in Fig. 7. This typical time-based GC profile illustrates the time-dependent composition of the liquid product. Table 7 lists the chemical compounds in the liquid product with their chemical formula, chemical weight and concentration. It is illustrated that amount of aromatic hydrocarbons decreased from 53.7 to 0.3% and normal aliphatic hydrocarbons increased from 3.1 to 24.8% after 30 min of run time. In line with this, the gasoline fractions (C5–C12) decreased from 84.1 to 52.9%^45^. Table 7 clearly illustrates changes in yield of wax, n-alkene content, aromatic content and gasoline fractions at the end of the processing. Alkanes account for nearly 56.31% of these chemicals, alkenes for 29.71%, and alcohol accounts for 13.98%. The cracking of the precursor's C–C bond causes the production of alkanes. Similarly, alkene production is attributed to the breakdown of C–H and C–C bonds. It contained C_6_–C_12_ and heavy gasoline-range hydrocarbons of C_13+_ fractions^46^.

**Table 7:** Chemical composition of the liquid product.

No	Hydrocarbons	Chemical formula	Molecular weight	Area concentration %	Retention time
1	1-Heptadecane	C_17_ H_34_	238.5	0.3	18.118
2	1-Heptadecyne	C_17_ H_32_	236.4	2.6	20.155
3	1-Heptadecamiene	C_17_ H_32_	236.4	0.3	23.218
4	n-Nonane	C_9_H_20_	128.9	15.5	25.134
5	Myristoleic acid	C_17_ H_34_	226.3	5.7	15.860
6	Cis-10 pentadecanoic acid	C_17_ H_32_O_2_	240.3	53.7	17.848
7	Methyle palmitate	C_17_ H_32_O_2_	270.4	0.1	19.455
8	Methyl heptadecanoate	C_9_H_20_O_2_	284.5	5.6	21.777
9	Methyl butyrate	C_5_ H_10_	102.1	0.9	2.260
10	Methyl hexanoate	C_7_ H_14_	130.1	0.9	2.433
11	Methyl tridecanoate	C_14_ H_28_	228.3	1.4	8.107
12	Methyl myristate	C_15_H_30_O_2_	242.4	11.0	11.227
13	Myristoleic acid	C_14_H_26_O_2_	226.3	3.1	15.600
14	Methyl palmitoleate	C_17_ H_32_O_2_	268.4	20.2	20.520
15	Methyl heptadecanoate	C_18_ H_36_O_2_	284.5	24.8	22.527
16	Cis-10-heptadedecanoic acid	C_17_H_32_	268.4	8.0	23.853
17	Trans-9-elaidic acid	C_18_H_32_	282.4	2.1	27.640
18	Cis-9-oleic acid	C_18_H_36_	282.5	10.7	29.307

**Figure 8:**
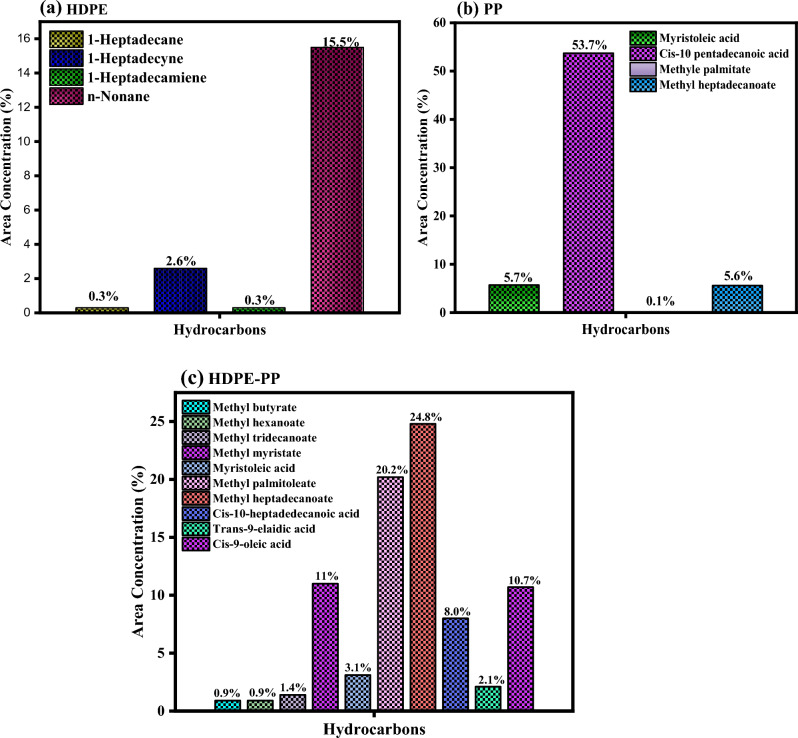
Graphical representation of components of oil from microwave pyrolysis of plastic: (**a**) HDPE, (**b**) PP, and (**c**) HDPE-PP.

In terms of chemical makeup, the products obtained using microwave pyrolysis were remarkably similar to those obtained through thermal pyrolysis. During microwave pyrolysis of plastic, a reduction in the catalytic activity of ZSM-5 was noticed; specifically, the catalytic activity was judged lowered after 30 min. Catalytic pyrolysis gave a higher liquid yield of 48.9% compared to 40.2% from thermal pyrolysis. Similarly, very little wax (1.2%) was found compared to 15.7% wax from thermal pyrolysis^47,48^. The aromatic percentage of the liquid product was 45%, which was significantly higher than the aromatic content of thermal pyrolysis, which was 18.6%. Similarly, microwave pyrolysis produced better yield of isomerized aliphatic (24.6%) compared to 10.4% from thermal pyrolysis and C5–C12 gasoline fractions of 73.5% compared to 54.3% from thermal pyrolysis. However, n-alkene content was much lower from microwave pyrolysis than thermal pyrolysis (12.8 vs. 44.0%). This is consistent with the catalyst's well-known catalytic activity in cracking, aromatization and isomerization due to its balanced mix of acidity, microporous structure, and shape selectivity^49,50^. The graphical representation of the area concentration of hydrocarbons is shown in Fig. 8.

## Conclusions

Polypropylene and high-density polyethylene were decomposed through in-situ catalytic Microwave pyrolysis using a ZSM-5 catalyst. The activated carbon was employed to absorb microwaves and then release heat to plastic for the pyrolysis into liquid and solid products. This process aimed to synthesize gasoline-range liquid fuel, hydrogen gas, and solid residue as CNTs. The highest efficiency of hydrogen gas (126.9 mmol/g) was analyzed through gas analyzer. The TGA analysis of plastic waste HDPE, PP and mixture of HDPE and PP showed the thermal degradation of plastic into solid residue at temperatures ranging from 3400 to 650 °C. The outer diameter of CNTs ranged from 30 to 93 nm from HDPE, 25 nm to 93 nm from PP and 30 nm to 54 nm for HDPE-PP mixure. The mixed waste produced the narrowest diameter CNTs. The structures were also more developed with uniform morphology than pure HDPE and PP. The catalyst particles are also smaller and lower in number in CNT structures from mixed plastic waste. FTIR profiles of liquid products of pyrolysis of PP, HDPE, and mixture of PP and HDPE revealed that liquid oil contained hydrocarbons with C–H bending of alkanes, alkenes, and alkyl group respectively. The gas chromatography analysis of liquid fuel revealed that oil fuel contained gasoline range hydrocarbon range of C_6_–C_12_ and heavy hydrocarbon C_13+_ fractions. The X-ray diffraction analysis of CNTs synthesized from plastic waste showed that at (002) diffraction peak, the high intensity and less FWHM value of CNTs have more hexagonal crystalline structure as compared to other diffraction peaks and intensities. The STEM analysis of CNTs illustrated tubular-like structural morphologies of carbon nanotubes synthesized from pyrolyzed waste plastic.

## Data Availability

The data reported in this paper is available from the corresponding authors on a reasonable request.
